# Plant canopy may promote seed dispersal by wind

**DOI:** 10.1038/s41598-021-03402-9

**Published:** 2022-01-07

**Authors:** Xuanping Qin, Wei Liang, Zhimin Liu, Minghu Liu, Carol C. Baskin, Jerry M. Baskin, Zhiming Xin, Zhigang Wang, Quanlai Zhou

**Affiliations:** 1 Institute of Applied Ecology, Chinese Academy of Sciences, Shenyang, 110016 China; 2 Zaozhuang University, Zaozhuang, 277101 China; 3 Experimental Center of Desert Forestry, Chinese Academy of Forest, Dengkou, 015200 China; 4 Department of Biology, University of Kentucky, Lexington, KY 40506 USA; 5 Department of Plant and Soil Sciences, University of Kentucky, Lexington, KY 40546 USA

**Keywords:** Ecology, Conservation biology

## Abstract

Seed dispersal has received much research attention. The plant canopy can intercept diaspores, but the effect of the plant canopy (the aboveground portion of a plant consisting of branches and leaves) on dispersal distance has not been explored empirically. To determine the effect of plant canopy on seed dispersal distance, a comparison of diaspores falling through open air and through plant canopy was made in a wind tunnel using three wind speeds and diaspores with various traits. Compared with diaspores falling through open air, the dispersal distance of diaspores falling through plant canopy was decreased or increased, depending on wind speed and diaspore traits. When falling through a plant canopy, dispersal distance of diaspores with thorns or those without appendages was promoted at low wind speed (2 m s^−1^), while that of diaspores with low wing loading (0.5 mg mm^−2^) and terminal velocity (2.5 m s^−1^) was promoted by relatively high (6 m s^−1^) wind speed. A plant canopy could increase seed dispersal distance, which may be due to the complicated updraft generated by canopy. The effect of maternal plants on seed dispersal regulates the distribution pattern and the species composition of the community.

## Introduction

Seed/diaspore dispersal can influence the spatial pattern and dynamics of a plant species at the population and metapopulation levels^1–5^, and it is an important research topic^6,7^. Seeds can be dispersed in multiple ways, and, on average, 10–30% of seeds and up to 70% of the plant species in temperate plant communities are more conductive to wind dispersal^8^. Any seed will be affected by wind^9^, which may further affect seed dispersal. Wind dispersal of diaspores occurs in all types of vegetation^3^, Wind can promote lateral dispersal speed of diaspores, and diaspore dispersal time/distance can be prolonged by strong horizontal wind^2,10^. The common wind levels in open xerophytic forest are 3–7 of Beaufort scale, occasionally can reach 8 levels, and wind speeds above 8 levels are rare^8^. Since wind speed and direction are variable, it is difficult to collect data on diaspore dispersal by wind in the field^11^. Thus, wind tunnels make it possible to conduct controlled experiments on the effects of wind on diaspore dispersal^12–15^.

Dispersal distance means how far a seed is moved away from the mother plant^3^, which affects the distribution pattern of seeds^10,16,17^. The pattern of diaspore dispersal will determine if the new plant becomes established in a habitat where the level of competition is low enough to be favorable for establishment and growth or if there is a high level of competition^17^. Thus, dispersal of diaspores may increase regional biodiversity^18^, and it can affect the management of weeds and endangered species^19^. Research on seed dispersal distance can facilitate an accurate prediction of population dynamics and distribution and improve the theoretical basis for vegetation restoration and biodiversity conservation.

Seeds are often the diaspores that are dispersed^9^, but the dispersed diaspores of angiosperms can be fruits or fruits plus appendages such as the bracts, perianth or parts of plants, tumbleweeds dispersal through whole plants^3,20–22^. The type of diaspore appendages will affect the way of seed dispersal^23,24^, and the diaspores with hairy or wing will be subjected to greater updraft^9^, which is more conducive to wind dispersal. Winged diaspores often fall in a rotating manner through the air, and the speed at which the size of the wing decreases is significantly correlated^25^. Diaspore traits, such as the maximum speed when air resistance exerted on the seed is equal to its pull of gravity during free fall in motionless air (terminal velocity) and the ratio of mass to projected area (wing loading), are important indicators to evaluate seed wind dispersal^14^.

The assumption in most theoretical studies, especially models on diaspore dispersal distance, is that diaspores are released through open air, and these studies have considered the movement of diaspores from the release point^20,26,27^. Although Cousens and Rawlinson (2001) considered the shape of the canopy into the seed dispersal model, they did not explore the influence of the morphological properties of the diaspore^28^. Previous field studies indicated that shrub canopy can intercept diaspores with appendages (hair, samara, wing, balloon) during low wind speeds^29^, however, the effect of seeds passing through the canopy on their dispersal distance is still unclear^30–34^. Pounden et al.^35^ found that winged diaspores had significantly reduced dispersal following boles collision, the hair diaspores did not, but how the plant canopy (i.e. aboveground portion of a plant consisting of branches and leaves) affects other types of diaspores is still unknown^36,37^. So, we hypothesized that the plant canopy can reduce diaspore dispersal distance, which is controlled by diaspore traits and wind speed. To test our hypothesis, controlled experiments were conducted in a wind tunnel, using 29 species with different diaspore traits.

## Materials and methods

### Diaspore selection and trait measurements

To determine the relationship between diaspore traits and dispersal distance of diaspores passing through plant canopy versus through open air at different wind speeds, we selected diaspores of 29 plant species that differ in appendage type, mass, projected area, shape index, wing loading, and terminal velocity (Table 1), differences in traits of the selected species were not restricted by phylogeny, and each diaspore was only considered as a representation of its own morphological attributes. Firstly, diaspores with different types of appendages (samara, wing, thorn, hair and balloon) were selected, and then the gradient of the same appendage diaspores was set according to the quality.

**Table 1:** Explanation of related terms.

Terms	Explanation
Hair Diaspore	Diaspores partially or fully covered with hairs (trichomed)
Wing Diaspore	Flat diaspores with a common device for becoming airborne
Samara Diaspore	Spherical diaspores with a common device for becoming airborne
Balloon Diaspore	A layer of the seed coat is loose or seed may be surrounded by inflated parts
Thorn Diaspore	Diaspores with hard spines, which often attached to livestock and human bodies to dispersal
No Appendage Diaspores	Diaspores without appendage
Mass	The weight of the diaspore
Projected area	Horizontal projection area of the diaspore when it is placed naturally
Shape index	An index indicating the shape of the diaspore, The smaller the index, the closer to the spherical shape, the larger the closer to the flat
Wing loading	The ratio of mass to projected area
Terminal velocity	The constant falling velocity of a seed in still air

To facilitate seed dispersal investigation, diaspores with samaras, wings, thorns, and without appendage were lightly sprayed with red aerosol paint, while diaspores with hairs were colored using red water-based markers. The dyed diaspores were naturally air-dried and placed in plastic boxes to ensure the integrity of morphological structure. Diaspore traits are measured after dyed and dried.

Twenty intact diaspores of each type were selected the same species for measurements of length, width, and thickness of each diaspore were measured with Vernier caliper (0.01 mm accuracy). Diaspores shape index (Vs) was calculated using an equation we developed from ideas in Thompson et al. (1993).

$$ V_{s} = \frac{{\mathop \sum \nolimits \left[ {x_{i} - \frac{{\mathop \sum \nolimits x_{i} }}{3}} \right]^{2} }}{N}, $$

where *N* = 3, $$x_{1} = \frac{{{\text{Length}}}}{{{\text{Length}}}}$$, $$x_{2} = \frac{{{\text{Width}}}}{{{\text{Length}}}}$$, and $$x_{3} = \frac{{{\text{Height}}}}{{{\text{Length}}}}$$.

Diaspore mass was determined using an electronic balance (0.0001 g), mass range of 29 diaspores were 1.12–316 mg. The projected area of diaspore was scanned with a digital scanner, and measured with analysis software (Motic Image Plus 2.0, Motic China Group Co., Xiamen, China), and project area range of 29 diaspores were 5–604 mm^2^. The wing loading was calculated as seed mass divided by projected area^38,39^, and wing loading range of 29 diaspores were 0.04–2.1 mg mm^−2^. Terminal velocity was measured with a camera described by Zhou et al. (2020), and terminal velocity range of 29 diaspores were 0.7–40 m s^−1^(Table 2).

**Table 2:** Diaspore traits of the 29 study species (mean ± SE).

Species	Abbreviation	Appendage type	Mass (mg)	Projected area (mm^2^)	Shape index	Wing loading (mg mm^−2^)	Terminal velocity (m s^−1^)
*Atriplex canescens*	Ac	Samara	31.35 ± 8.569	77.276 ± 13.293	0.007 ± 0.005	0.409 ± 0.093	2.485 ± 0.228
*Caligonum leucocladum*	Cl	Samara	149.975 ± 25.142	218.669 ± 26.005	0.003 ± 0.003	0.686 ± 0.08	3.098 ± 0.138
*Caligonum rubicundum*	Cr	Samara	52.095 ± 4.929	166.598 ± 16.548	0.002 ± 0.003	0.314 ± 0.028	2.255 ± 0.164
*Haloxylon ammodendron*	Ha	Samara	6.776 ± 1.383	36.839 ± 4.368	0.063 ± 0.022	0.186 ± 0.04	1.685 ± 0.353
*Sympema regelii*	Sr	Samara	7.78 ± 1.891	54.844 ± 12.48	0.082 ± 0.021	0.145 ± 0.031	1.383 ± 0.225
*Zygophyllum xanthoxylon* (three-winged)	Zx	Samara	163.47 ± 43.58	558.871 ± 102.138	0.013 ± 0.007	0.291 ± 0.045	1.959 ± 0.244
*Acer saccharum*	As	Wing	35.799 ± 5.255	197.827 ± 20.144	0.16 ± 0.002	0.181 ± 0.015	0.78 ± 0.066
*Althaea rosea*	Ar	Wing	16.935 ± 0.613	44.744 ± 1.626	0.119 ± 0.013	0.379 ± 0.02	2.728 ± 0.473
*Ferula bungeana*	Fb	Wing	21.225 ± 2.766	50.52 ± 8.331	0.129 ± 0.008	0.428 ± 0.075	2.74 ± 0.223
*Syzygium aromaticum*	Sa	Wing	9.905 ± 1.807	25.481 ± 2.892	0.152 ± 0.011	0.394 ± 0.086	2.428 ± 0.27
*Ulmus pumila*	Up	Wing	10.22 ± 2.421	242.738 ± 34.34	0.17 ± 0.011	0.043 ± 0.012	0.908 ± 0.106
*Zygophyllum xanthoxylon* (disc)	Zxd	Wing	90.685 ± 18.334	603.932 ± 83.549	0.142 ± 0.016	0.152 ± 0.031	1.646 ± 0.213
*Calligonum alaschanicum*	Ca	Thorn	44.42 ± 10.625	73.215 ± 16.064	0.007 ± 0.004	0.615 ± 0.122	3.285 ± 0.116
*Lappula intermedia*	Li	Thorn	6.775 ± 1.664	11.156 ± 1.702	0.004 ± 0.003	0.607 ± 0.12	2.225 ± 0.441
*Psilopeganum sinense*	Ps	Thorn	1.065 ± 0.496	5.125 ± 0.446	0.067 ± 0.023	0.211 ± 0.105	1.844 ± 0.416
*Tribulus terrestris*	Tt	Thorn	26.345 ± 13.79	24.244 ± 5.742	0.016 ± 0.008	1.167 ± 0.74	2.714 ± 0.239
*Xanthium sibiricum*	Xs	Thorn	74.66 ± 21.572	45.319 ± 3.998	0.027 ± 0.004	1.677 ± 0.576	3.729 ± 0.213
*Catalpa ovata*	Co	Hair	5.005 ± 1.227	73.216 ± 16.911	0.194 ± 0.004	0.069 ± 0.015	1.078 ± 0.173
*Clematis hexapetala*	Ch	Hair	4.095 ± 1.023	79.993 ± 21.214	0.083 ± 0.018	0.056 ± 0.023	1.008 ± 0.242
*Echinops gmelinii*	Eg	Hair	9.115 ± 1.86	40.598 ± 4.212	0.006 ± 0.004	0.227 ± 0.055	2.195 ± 0.225
*Reaumuria trigyna*	Rt	Hair	38.975 ± 9.163	111.218 ± 27.21	0.012 ± 0.008	0.482 ± 0.65	2.024 ± 0.257
*Carex lehmanii*	Cle	None	5.165 ± 0.403	8.218 ± 0.635	0.101 ± 0.005	0.631 ± 0.057	1.258 ± 0.461
*Euonymus maackii*	Em	None	40.865 ± 10.544	20.209 ± 2.554	0.041 ± 0.009	2.037 ± 0.526	2.47 ± 0.252
*Messerschmidia sibirica*	Ms	None	66.6 ± 7.452	34.095 ± 3.187	0.01 ± 0.006	1.961 ± 0.234	3.457 ± 0.262
*Nitraria tangutorum*	Nt	None	29.935 ± 7.056	20.489 ± 3.345	0.071 ± 0.015	1.455 ± 0.197	2.825 ± 0
*Platycladus orientalis*	Po	None	22.405 ± 10.257	14.758 ± 1.586	0.062 ± 0.011	1.537 ± 0.726	3.01 ± 0.39
*Thermopsis lanceolata*	Tl	None	18.185 ± 3.573	9.322 ± 0.781	0.019 ± 0.003	1.956 ± 0.378	2.858 ± 0.371
*Sect.arenicola*	***Sa***	Balloon	18.695 ± 6.681	69.533 ± 12.684	0.03 ± 0.012	0.269 ± 0.078	1.66 ± 0.165
*Sphaerophysa salsula*	Ss	Balloon	315.95 ± 56.919	406.716 ± 61.464	0.061 ± 0.013	0.779 ± 0.104	3.109 ± 0.499

### Wind speed control

A wind tunnel (with a test section 2 m high × 2 m wide × 20 m long) was used to control wind speed. Wind speed was monitored inside the tunnel with a Pitot tube (160-96, Dwyer Instruments, Inc., Indiana, USA) connected with a Magnesense II Differential Pressure Transmitter (MS2-W102-LCD, Dwyer Instruments Inc., Indiana, USA) (Liang et al*.* 2020). In this study, wind speeds (measured 1 m above the flat sand surface) were set at 2, 4 and 6 m s^−1^, and they correspond to categories 3–5 of meteorological wind measurements, which is basically close to the common range of wind speeds under natural conditions (Mather, 1987). The underlying surface is set with a flat fixed sand surface in this experiment.

### Model plant canopy setting

**Figure 1:**
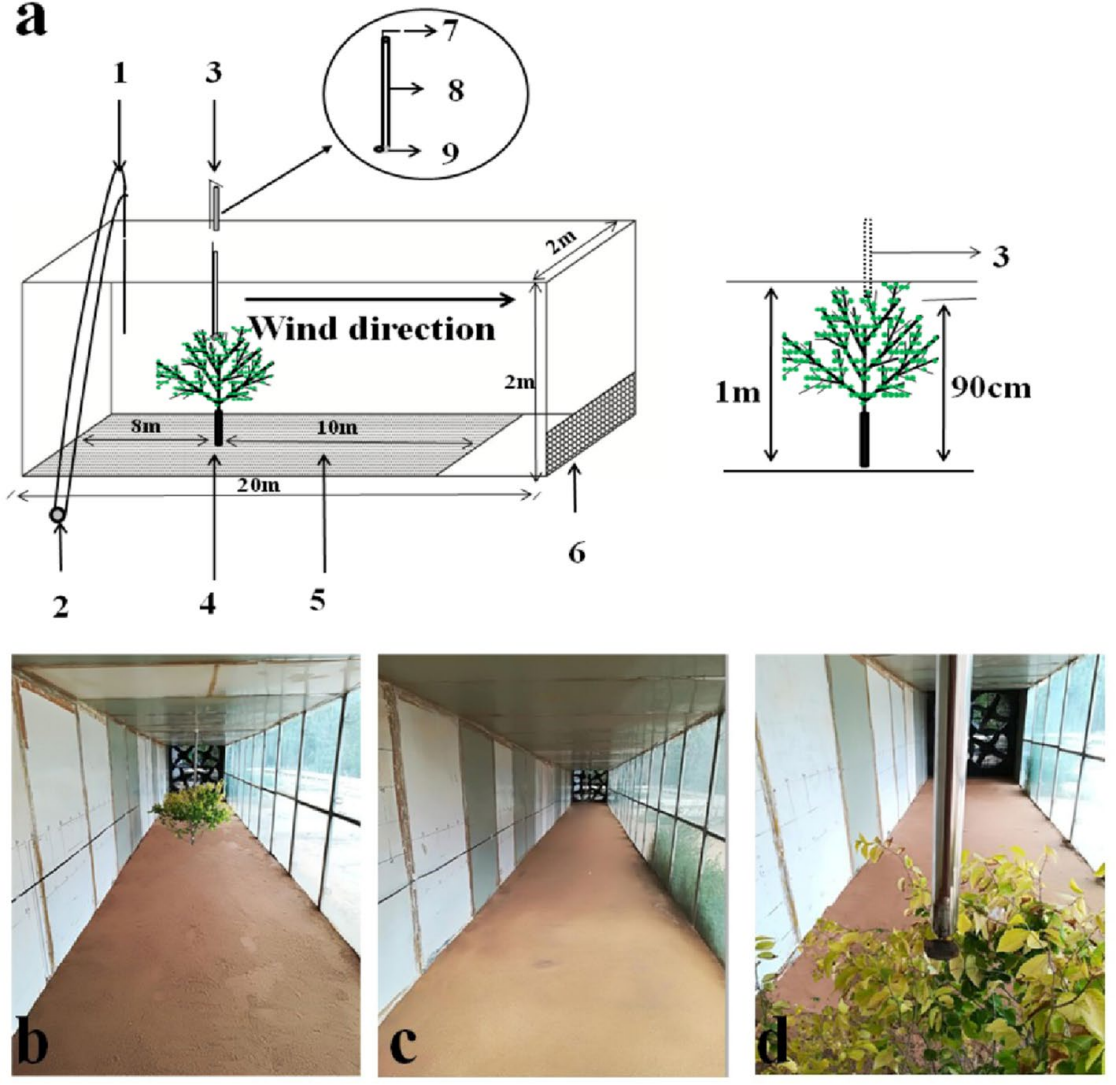
Diagram of wind tunnel used in the study. (**a**) 1, Pitot tube; 2, differential pressure transmitter; 3, diaspore release device (seed release point was 90 cm above the ground, and placed 10 cm in the center of the canopy); 4, leafy plant (plant height was 1 m); 5, experiment section; 6, diaspore-blocking net; 7, switch; 8, steel tube; 9, bottom flap. (**b**) Inside view of the wind tunnel showing position of the plant. (**c**) Inside view of the wind tunnel without the plant. (**d**) Close-up view of plant inside the wind tunnel showing position of diaspore release device. The photograph in this figure was taken by Xuanping Qin.

A young tree (sapling) of *Ulmus parvifolia* was selected for use as the model canopy because it has a high density of branches and many leaves. Further, this species is relatively easy to transplant and tolerant of high wind speed. Plant height was 1 m (Fig. 1), all leaves were present, and size of canopy was 1.0 m high × 1.2 m wide. The model plant selected was only used to explore the effect of canopy on seed dispersal distance and was not related to the maternal plants of the experimental diaspores. The model sapling was transplanted (transplanted with roots to ensure plant survival, and the root part is fixed with sand to keep the underlying surface flat) from the field to the front end of the wind tunnel test section,8 m away from the power section and in the middle of the two walls of the wind tunnel. The model plant grew well throughout the experiment.

### Diaspore release

To standardize our studies of the dispersal of diaspores through the canopy, we set the diaspore release point as the center of the canopy, 10 cm below the top of the canopy and 90 cm from the soil surface. A 4 cm diameter stainless steel tube was used as the release device. The release device was inserted into the canopy from the top of the wind tunnel. Diaspores were released from the upper part of the steel tube (which was controlled by a bottom flap to ensure that initial release rate was zero) when the target wind speed was reached. Five replicates of 20 diaspores of each type were used for each wind speed.

### Effect of wind speed on diaspore dispersal through plant canopy

Five replicates of 20 diaspores of each type were used for each wind speed . The 20 diaspores were released simultaneously from the release device through the plant canopy, when the target wind speed was reached (2, 4 or 6 m s^−1^). We turned off the wind tunnel after 2 s of diaspore dispersal. The horizontal (dispersal) distance that each of the 20 diaspores was moved from the release point was measured. Mean dispersal distance was used to represent dispersal ability.

The control was the release of diaspores at a height of 90 cm from the ground through air at the three wind speeds, i.e. after the plant was removed from the wind tunnel. Diaspore dispersal distance was measured after the wind speed was maintained for 2 s.

### Statistical analysis

The difference in the dispersal distance of each type of diaspore passing through the canopy versus through open air at different wind speeds was compared. Redundancy analyses (RDA) based on correlation matrices of diaspore dispersal distance and explanatory factors were conducted using Canoco 5.0 (version 5.0, Microcomputer Power, Ithaca, NY, USA; Tackenberg, 2003). We analyzed the contribution of diaspore mass, projected area, wing loading, terminal velocity, and shape index to dispersal distance and the relationship between the plant's effect on dispersal distance and diaspore traits. Statistical analyses were conducted using IBM SPSS Statistics 22.0 (IBM Corporation 1989, 2013, USA), and plots were drawn by Origin Pro 8.5 (Origin Lab Corporation 1991–2010, USA).

## Results

### Influence of plant canopy on diaspore dispersal distance at different wind speeds

**Figure 2:**
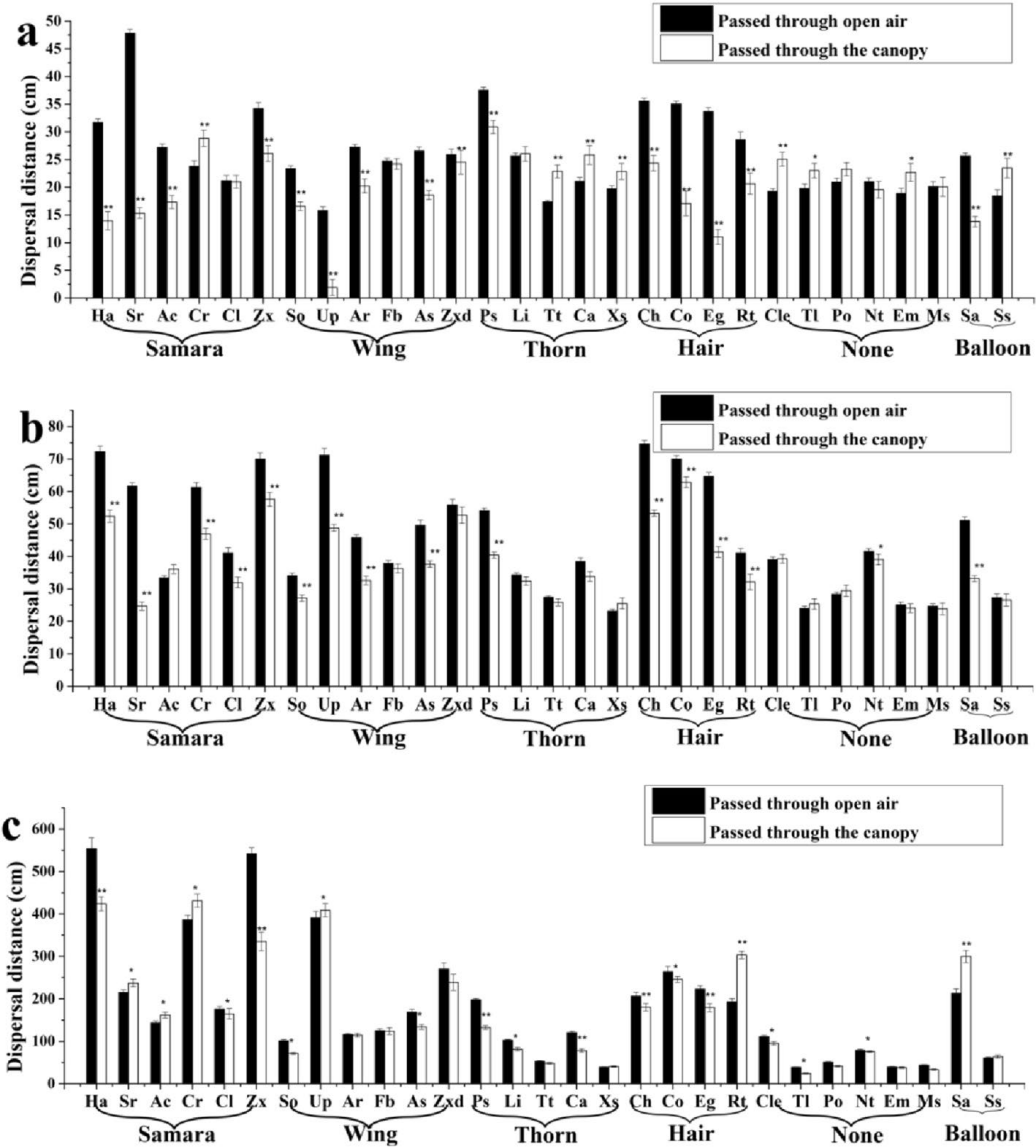
Dispersal distance of diaspores of the 29 species that passed through plant canopy or through open air at three wind speeds. (**a–c**) wind speed was 2, 4, and 6 m s^−1^, respectively. * (*P* < 0.05) and ** (*P* < 0.01) indicate significant differences between dispersal distance after passing through plant versus open air.

Plant canopy either increased or decreased diaspore dispersal distance, depending on diaspore traits and wind speed. Compared with dispersal through open air, dispersal distance of nine diaspore types (1 samara (of 7 tested), 3 thorn (of 5 tested), 4 without an appendage (of 7 tested), and 1 balloon (of 2 tested)) was significantly promoted by the plant canopy at a wind speed of 2 m s^−1^. Dispersal distance of diaspores with a thorn or those with no appendage was more likely to be promoted by the plant canopy than that of the other kinds of diaspores at a wind speed of 2 m s^−1^. However, dispersal distance of diaspores with a wing and those with hair was decreased by the plant canopy. The dispersal distance of all types of diaspore passed through the canopy were not significantly increased compared with that of diaspore passed through the open air when the wind speed was 4 m s^−1^. Dispersal distance of six diaspore types (3 samara (of 7 tested), 1 wing (of 7 tested), 1 hair (of 4 tested), and 1 balloon (of 2 tested)) was significantly increased by the plant canopy at a wind speed of 6 m s^−1^, and dispersal distance of diaspores with a thorn or those with no appendage was decreased by the plant canopy. At a wind speed of 6 m s^−1^, dispersal of diaspores with a wing was increased more by the canopy than that of the other kind of diaspores, and dispersal distance for diaspores without an appendage was significantly decreased (Fig. 2).

### Influence of diaspore traits on dispersal distance by wind

**Figure 3:**
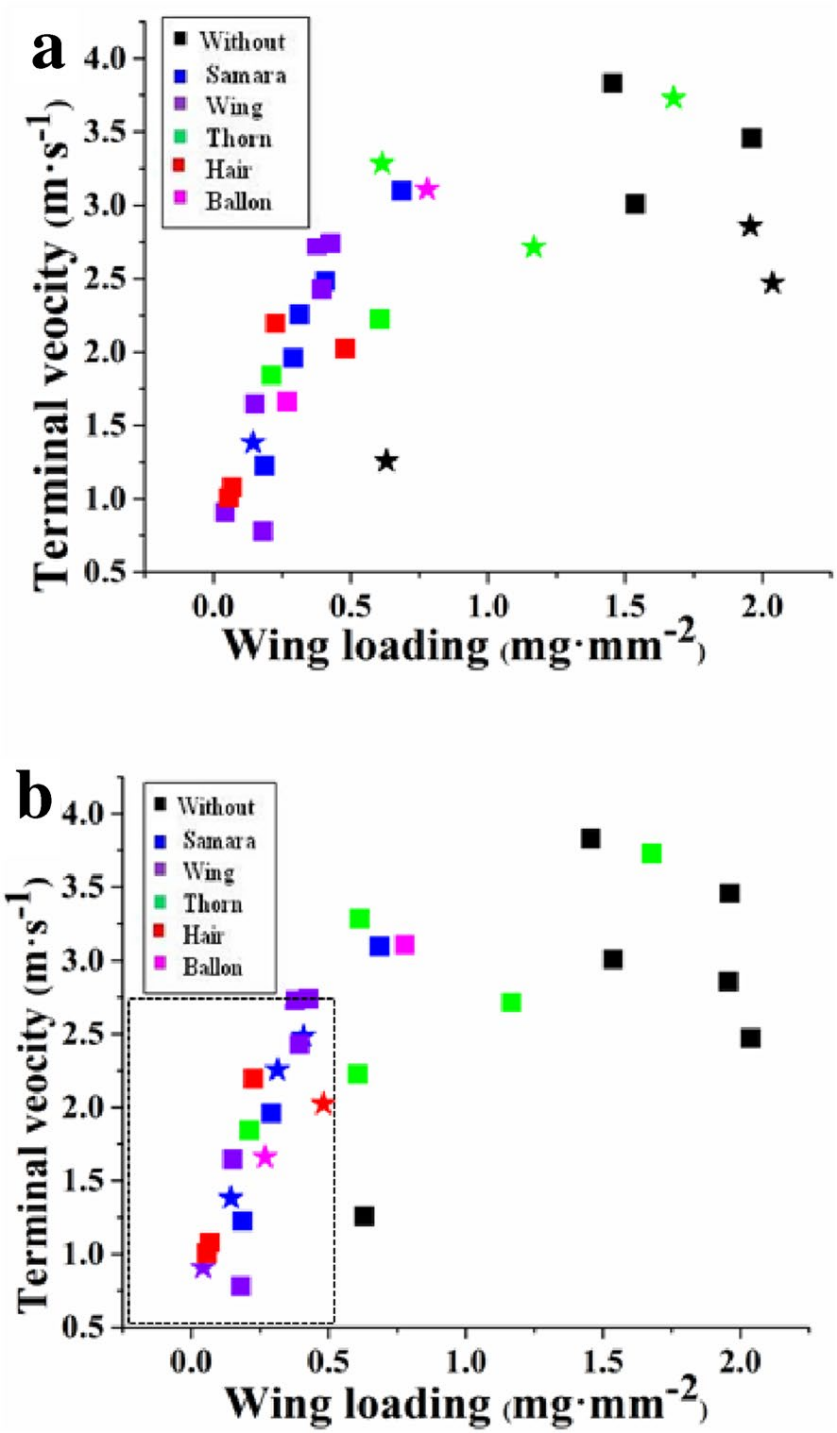
The effect of plant canopy on the dispersal distance of different diaspores. (**a, b**) Wind speed 2 and 6 m s^−1^. Squares represent diaspores for which plant canopy decreased dispersal distance, and star represent diaspores for which plant canopy increased dispersal distance. Colors represent diaspore with different appendage: black without appendage; blue, a samara; violet, with wing; green, with thorn; red, with hair; pink, with balloon.

The contribution (percentage) of diaspore traits to dispersal distance increased with wind speed (Table 3). Wing loading and terminal velocity had negatively correlations with diaspore dispersal distance for diaspores released through open air and through the plant canopy at wind speeds of 4 and 6 m s^−1^, and wing loading was more important than terminal velocity for dispersal distance. However, wing loading and terminal velocity did not have a significant effect on diaspore dispersal distance through the plant canopy at a wind speed of 2 m s^−1^. Neither shape index, projected area nor mass had a significant effect on diaspore dispersal distance either through the plant canopy or through open air at any of the three wind speeds. At a wind speed of 6 m s^−1^, the plant canopy increased dispersal-distance of diaspores with a small wing loading (≤ 0.5 mg mm^−2^) and terminal velocity (≤ 2.5 m s^−1^), and samaras had the greatest dispersal distance followed by diaspores with wing, hair, and balloon. The plant canopy decreased dispersal distance of diaspores with high wing loading and high terminal velocity (Fig. 3).

**Table 3:** The relationship between diaspore traits and dispersal through open air and through plant canopy.

Dispersal treatment	Wind speeds(m s^−1^)	E	WL	TV	SI	PA	MS
Release through open air	2	43.70	36.0**0	23.50**	0.90	< 0.01	6.00
	4	73.20	69.20**	51.80**	12.10	0.09	0.04
	6	83.50	73.90**	46.30**	4.90	17.20*	0.80
Release through canopy	2	17.40	7.20	12.60	12.20	< 0.01	4.40
	4	57.90	45.00**	34.00**	12.80*	15.80*	1.40
	6	70.60	53.90**	48.50**	1.50	11.40	0.00

E, percentage explained by diaspore traits; PA, projected area (mm^2^); WL, wing loading (mg mm^−2^); TV, terminal velocity (m s^−1^); SI, shape index; MS, diaspore mass (mg); *0.01 < p < 0.05; ** p < 0.01.

## Discussion

Our study indicated that compared to dispersal through open air, diaspores with thorns or without an appendage were more strongly deflected by the canopy at low wind speed, thus their dispersal distance was also more likely to increase. Previous study found that the plant canopy had little effect on interception of diaspores with thorns and without appendages^29^, but it will disperse farther after passing through the canopy at low wind speed. Other diaspores (samara, hair, wing, and balloon) will be more easily intercepted by plant canopy at low wind speed^29^, and thus their dispersal distance was more likely to be decreased. Our study showed that the dispersal distance of samara diaspores were more easily increased by plant canopy at a higher wind speed than dispersal through air (Fig. 2), this may because samara diaspores were subjected to a strong updraft, The effect of wind on seed dispersal distance is complex in nature, resulting in different vertical and horizontal components of air flow including turbulence^10,40,41^, and complex updrafts potentially increasing wind dispersal within a canopy^42^.

The promotion of dispersal distance of some diaspores falling through the plant canopy (Fig. 3) is contrary to the branch interception of diaspores^43,44^. Increased dispersal distance may due to induction of an updraft by wind hitting the plant canopy or by change in direction of diaspores that collide with a plant part can change the original dispersal trajectory and increase dispersal distance. Wind conditions can be altered when wind passes through a plant canopy, such as by the generation of an updraft, increasing of vertical wind speed, and reducing of horizontal speed^39,45,46^. Low horizontal wind speed will decrease diaspore dispersal distance, but an updraft or vertical wind speed can increase dispersal time, thereby increasing diaspore dispersal distance. Thus, the plant canopy can increase or decrease diaspore dispersal distance compared to diaspores passing through open air at the same wind speed.

Tackenberg et al.^47^ found that updrafts are important for long-dispersal of dandelion in an open meadow environment. Our results showed that plant canopy not only promoted the dispersal of hairy diaspores, but also promoted the dispersal of diaspores with samara, wing, and balloon, which might be because of localised updrafts and eddies caused by plant canopy. Variety of canopy types will cause differences in turbulence, which may further affect the distance of seed wind dispersal. The canopy of branch and leaf density may produce complex updrafts, therefore, we speculate that a dense canopy of branches and leaves is more likely to promote seed wind dispersal, and wind-borne seeds are easier to dispersal over long distances in forests than in deserts, of course, this needs further research to confirm.

Diaspore dispersal distance is strongly correlated with wing loading and terminal velocity^3,48,49^. Our study indicated that the dispersal distance of diaspores with small wing loading and terminal velocity can be promoted by the plant canopy at high wind speed but not at a low wind speed (Fig. 3). This is because diaspores with low wing loading and terminal velocity are easily accelerated laterally by the drag of the wind following a collision with a solid object during high wind speed. Diaspores that are dispersed by wind generally have a smaller terminal velocity and wing loading, this means that wind-borne species are distributed more widely in vegetation-covered areas than in open landscapes.

In conclusion, a plant canopy may increase dispersal of diaspores with thorns or those without appendages during low wind speed, and dispersal of diaspores with small wing loading and terminal velocity at high wind speeds. An estimation of the plant canopy effect on diaspore dispersal distance can improve the accuracy of the assessment of dispersal distance of a cohort of diaspores in the real world. Accordingly to our results, we can predict that diaspores with small terminal velocity and wing loading are easier to dispersal long distances under hurricane or storm conditions.

## Data Availability

The data presented in this study are available on request from the corresponding author.
